# Ti_3_C_2_ nanosheet-induced autophagy derails ovarian functions

**DOI:** 10.1186/s12951-024-02495-4

**Published:** 2024-05-12

**Authors:** Limei Yang, Zhiting He, Le Hu, Hongyu Tang, Yanqing Geng, Qiaoyan Tan, Yue Zhang, Yixian Wen, Wei Wu, Huayan Gu, Xueqing Liu

**Affiliations:** 1 Department of Obstetrics and Gynecology, Women and Children’s Hospital of Chongqing Medical University, No. 120 Longshan Road, Yubei District, Chongqing, 401147 China; 2 Chongqing Municipal Health Commission Key Laboratory of Perinatal Medicine, Chongqing, 400016 China; 3 Joint International Research Laboratory of Reproductive and Development, Department of Reproductive Biology, School of Public Health and Management, Chongqing Medical University, No. 1 Yixueyuan Road, Yuzhong District, Box 197, Chongqing, 400016 China; 4 Department of Obstetrics and Gynecology, Gansu Provincial Clinical Research Center for Gynecological Oncology, the First Hospital of Lanzhou University, Lanzhou, Gansu 730000 China; 5 Department of Pediatrics, Women and Children’s Hospital of Chongqing Medical University, Chongqing, 401147 China; 6 Prenatal Diagnosis Center, Women and Children’s Hospital of Chongqing Medical University, Chongqing, 401147 China; 7 College of Basic Medicine, Chongqing Medical University, Chongqing, China; 8 Senior Department of Ophthalmology, 3rd Medical Center of Chinese PLA General Hospital, Beijing, 100039 China

**Keywords:** Ti_3_C_2_ nanosheets, Autophagy, PI3K/AKT/mTOR, Hormone, Follicle

## Abstract

**Background:**

Two-dimensional ultrathin Ti_3_C_2_ (MXene) nanosheets have gained significant attention in various biomedical applications. Although previous studies have described the accumulation and associated damage of Ti_3_C_2_ nanosheets in the testes and placenta. However, it is currently unclear whether Ti_3_C_2_ nanosheets can be translocated to the ovaries and cause ovarian damage, thereby impairing ovarian functions.

**Results:**

We established a mouse model with different doses (1.25, 2.5, and 5 mg/kg bw/d) of Ti_3_C_2_ nanosheets injected intravenously for three days. We demonstrated that Ti_3_C_2_ nanosheets can enter the ovaries and were internalized by granulosa cells, leading to a decrease in the number of primary, secondary and antral follicles. Furthermore, the decrease in follicles is closely associated with higher levels of FSH and LH, as well as increased level of E_2_ and P_4_, and decreased level of T in mouse ovary. In further studies, we found that exposure toTi_3_C_2_ nanosheets increased the levels of Beclin1, ATG5, and the ratio of LC3II/Ι, leading to autophagy activation. Additionally, the level of P62 increased, resulting in autophagic flux blockade. Ti_3_C_2_ nanosheets can activate autophagy through the PI3K/AKT/mTOR signaling pathway, with oxidative stress playing an important role in this process. Therefore, we chose the ovarian granulosa cell line (KGN cells) for in vitro validation of the impact of autophagy on the hormone secretion capability. The inhibition of autophagy initiation by 3-Methyladenine (3-MA) promoted smooth autophagic flow, thereby partially reduced the secretion of estradiol and progesterone by KGN cells; Whereas blocking autophagic flux by Rapamycin (RAPA) further exacerbated the secretion of estradiol and progesterone in cells.

**Conclusion:**

Ti_3_C_2_ nanosheet-induced increased secretion of hormones in the ovary is mediated through the activation of autophagy and impairment of autophagic flux, which disrupts normal follicular development. These results imply that autophagy dysfunction may be one of the underlying mechanisms of Ti_3_C_2_-induced damage to ovarian granulosa cells. Our findings further reveal the mechanism of female reproductive toxicity induced by Ti_3_C_2_ nanosheets.

**Supplementary Information:**

The online version contains supplementary material available at 10.1186/s12951-024-02495-4.

## Graphical Abstract

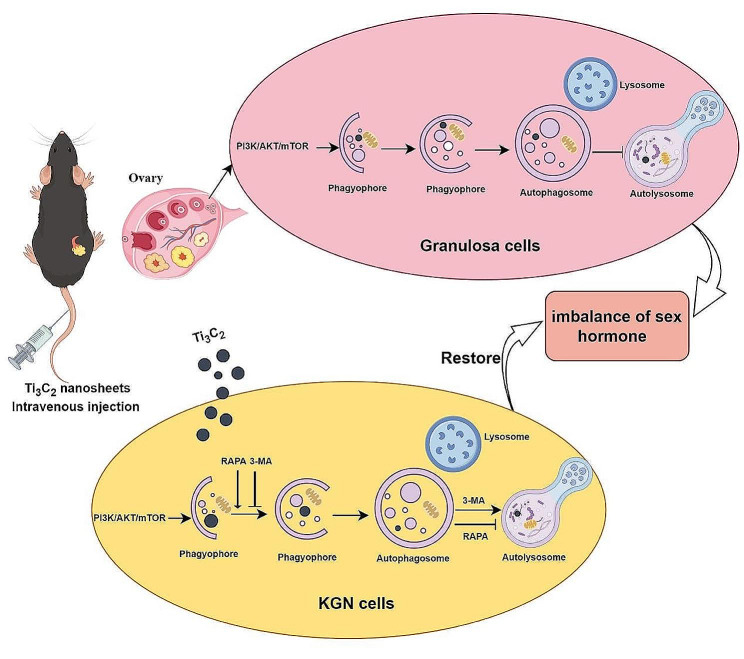

## Introduction

In recent years, the decline in women’s fertility has become a serious issue, with ovulation disorders being one of the main contributing factors [1]. Apart from social shifts such as delayed childbearing and increased use of contraception by women, environmental exposure is also regarded as an important factor. Numerous in vitro, in vivo, and epidemiological studies have demonstrated that exposure to environmental pollutants including endocrine-disrupting chemicals, plastics, cosmetics, and hygiene products can disrupt hormone balance, affect women’s follicular development, cause reproductive disorders and lower fertility rates [2]. The ovary serves as a vital organ with diverse functions, including the synthesis of sex steroid hormones, promotion of follicular development, facilitation of oocyte meiotic division, as well as supporting successful fertilization and embryo development [3]. Granulosa cells (GCs) are pivotal somatic cells derived from the sex cord that play a vital role in the development of follicles and maturation of oocytes. As the major source of estradiol (E_2_) and estrogens in response to follicle-stimulating hormone (FSH), granulosa cells interact closely with oocytes to promote their development, maturation, and ovulation. To put it differently, GC dysfunction usually leads to imbalances in reproductive hormone regulation, pathological follicle formation, as well as conditions such as polycystic ovary syndrome (PCOS) and premature ovarian failure (POF) [3–5].

The rapid development of nanotechnology has resulted in the extensive utilization of diverse nanomaterials in areas such as cosmetics, paints, medical products, and personal care items. As a consequence, human exposure to nanomaterials has significantly increased [6]. Recent studies have revealed that female exposure to nanomaterials may cause long-term adverse effects on ovaries. For instance, NPs can penetrate the protective barriers of follicular cells, granulosa cell layers, and the zona pellucida, thereby interfering with the development of immature oocytes [7]. Exposure to Cu NPs can directly harm ovaries, disrupting the enzymes involved in hormone production and resulting in reproductive dysfunction [8]. Previous research has suggested that excessive activation or inhibition of autophagic flux leading to decreased autophagic function may be a possible mechanism behind toxicity caused by nanoparticles [9, 10]. Autophagy is a conserved and protective cellular program that plays an essential role in maintaining cellular homeostasis in eukaryotic cells. It involves the degradation and recycling of unwanted or damaged proteins, organelles, and other intracellular components through the lysosomal degradation pathway [11]. Autophagy induction is primarily associated with cellular responses to oxidative stress, drug interventions, starvation, and nutrient limitations. Accumulating evidence suggests that in this process, Beclin1 acts as a scaffold protein to promote the initiation of autophagosome formation; ATG5 forms a complex with ATG12, for the membrane elongation and formation of autophagosomes; LC3 participates in the formation of autophagosomes and degradation of cargo; p62 (also known as SQSTM1) facilitates the degradation and clearance of waste material. Autophagy dysfunction, including impaired autophagic pathway initiation and incomplete removal of cellular waste, can lead to various metabolic disorders, cancer, and neurodegeneration [12, 13]. Furthermore, ovarian dysfunction has been linked to both autophagy induction and dysfunction. ZnO NPs can induce cytotoxicity in mature oocytes by activating autophagy and cell apoptosis through a cysteine-dependent mechanism [14]. Zhen Zheng et al. revealed that SNPs induce autophagy dysfunction through lysosomal impairment, leading to the blockage of autophagic flux, which in turn enhances apoptosis and results in follicular atresia in ovarian granulosa cells [15].

The new two-dimensional (2D) nanomaterial titanium carbide (MXene), which is composed of Ti_3_C_2_, has garnered a great deal of attention because of its exceptional physical and chemical characteristics. MXenes are transition metal carbides and nitrides with the structural formula M_*n*+1_X_n_T_x_ (*n* = 1–3), where M denotes a transition metal (e.g., Sc, Ti, Zr, Hf, V, Ta, Nb, Cr, or Mo); T is a surface functionality group, and X stands for carbides, nitrides, or carbonitrides [16]. This peak was extracted from the matching MAX phase by selectively etching the interlayer Al atoms in the transition metal carbide Ti_3_AlC_2_ [17]. Owing to their excellent biocompatibility, MXenes have opened up significant opportunities for development in various application fields, including antibacterial agents [18], drug delivery [19], biosensing [20], photothermal/photodynamic therapy [21], and wastewater treatment [22]. However, we must also note the existence of some negative effects. MXenes have a flat, sheet-like structure, which increases the surface area for interaction with the cell membrane, thereby enhancing the likelihood of cellular uptake. Previously, we found that exposure to Ti_3_C_2_ nanosheets resulted in abnormal female placental development and decreased male sperm motility [23–25]. Studies have shown that exposure to TiO_2_ NP results in decreased ovarian weight, accompanied by a reduction in primordial follicles, secondary follicles, antral follicles, and corpora lutea, along with an increase in atretic follicles [26, 27]. TiO_2_ NP induce ovarian tissue damage in mice leading to ovarian dysfunction through lipid peroxidation and hormonal imbalance [28]. To date, there has been limited experimental research on the reproductive safety of Ti_3_C_2_ nanosheets, and the impact of Ti_3_C_2_ nanosheets on ovarian function has not been studied.

The purpose of this study was to explore the impact of Ti_3_C_2_ nanosheets exposure on ovarian hormone production and to explore the underlying mechanisms involved. Our investigation involved C57BL/6J female mice and the KGN cell line. The outcomes of our research elucidated the detrimental effects of Ti_3_C_2_ nanosheets, which induce autophagy and impair autophagic flux, disrupting the endocrine balance within the ovaries. Notably, we discovered the potential involvement of the PI3K/AKT/mTOR signaling pathway in Ti_3_C_2_ nanosheet-induced autophagy. By filling gaps in knowledge regarding the reproductive toxicity of Ti_3_C_2_ nanosheets in females, our findings provide essential experimental evidence for evaluating their safety. Furthermore, this study has promising implications for the clinical application of this nanomaterial and offers valuable insights.

## Materials and methods

### Preparation and characterization of Ti_3_C_2_ nanosheets

The preparation and characterization methods used for the Ti_3_C_2_ nanosheets were consistent with our previous research [23–25].

### Animal model

Adult female mice (C57BL/6J, aged 8–10 weeks, 18–20 g) were purchased from the Animal Laboratory Center of Chongqing Medical University, and obtained approval from the Institutional Animal Care and Use Committee of Chongqing Medical University. All mice were acclimated for at least 7 days in an isolated animal room, maintained at a constant temperature of 22 ± 2 °C and humidity of 50%, following a 12-hour light–dark cycle and were also given with unlimited forage and water *ad libitum*.

Because Ti_3_C_2_ nanosheets have been studied as a possible photothermal agent for cancer therapy, we decided to use the lowest published dose (5 mg/kg) as the highest daily dose in this study [29]. In order to establish a safe dosage range for mice. 25% of the highest dose was designated as the lowest dose (1.25 mg/kg), and half of the highest dose (2.5 mg/kg) as the intermediate dose. We examined the estrous cycle of the mice employing the vaginal smear method. Mice in the diestrus stage were randomly divided into four groups of 5 mice in each group, and conduct multiple experiments, with a total of more than 80 mice. Ti_3_C_2_ nanosheets at doses of 1.25, 2.5, and 5 mg/kg/day body weight were administered intravenously to three experimental groups for three days, whereas saline injections were given to the control group. Ti_3_C_2_ nanosheets were dispersed in saline and had good dispersion. The concentrations of Ti_3_C_2_ nanosheets stock solution is 7.5 mg/mL and diluted in saline were 0.125 mg/mL, 0.25 mg/mL and 0.5 mg/mL, respectively. The volume of the Ti_3_C_2_ nanosheet suspension used for exposure was 0.1 mL/10 g body weight. On the fourth day, all mice were euthanized to collect ovarian tissues and serum for subsequent experiments.

### Estrous cycle detection

We followed earlier techniques [30]. Sterile cotton swabs moistened with saline were gently inserted into the mouse vagina to collect exfoliated cells. These cells were then smeared onto clean glass microscope slides and stained with Wright–Giemsa solution (Solarbio, China). In the diestrus phase, the characteristic feature is a moderate-to-low cellularity of predominantly neutrophils mixed with a smaller number of epithelial cells.

### Accumulation of Ti_3_C_2_ nanosheets in vivo detected by ICP‒MS

To reach the detection limit, ovarian tissues were collected from more than 15 mice, and more than 60 mg was weighed from each group. Ti_3_C_2_ nanosheet accumulation in the ovaries was quantified for Ti content using Inductively coupled plasma‒mass spectrometry (ICP‒MS) with a model 7800 instrument from Agilent Technologies, Inc., USA. The evaluation was conducted by Beijing Zhongkebaice Technology Co., Ltd.

### Transmission electron microscopy (TEM)

The collected mouse ovarian tissue was immediately immersed in a 2.5% glutaraldehyde solution at a pH of 7.4 for fixation. Then, the tissue was further fixed in 1% osmium tetroxide (OsO4) and dehydrated in epoxy propane. Following dehydration, 70 nm-thick ultrathin slices were gathered and dyed with lead citrate and uranyl acetate.

Following 24 h exposure to varying concentrations of Ti_3_C_2_ nanosheets, the KGN cells were washed with PBS for 3 min and subjected to trypsin solution digestion. The cell suspension was centrifuged at 1200 rpm for 10 min, after which the supernatant was discarded. To evaluate the samples, cells were collected and prefixed with a 2.5% glutaraldehyde solution. The samples were observed by Beijing Zhongkebaice Technology Co., Ltd.

### H&E staining and number of mature follicles

Fresh mouse ovarian tissues were fixed in 4% paraformaldehyde for four to six hours, dehydrated with a gradient of ethanol to remove water. And then embedded in paraffin to fix and protect the tissue structure. The tissue samples were sliced into 5 μm thick sections using a paraffin microtome. After that, Hematoxylin and eosin (HE) staining was applied to the slices, and the sections were observed using an Olympus microscope (BX40, Tokyo, Japan). Five typical glides were selected from each ovary. The interval between slides was set at > 200 μm to prevent duplicate counting of follicles.

### **Cell culture and treatment**

KGN cells, a human ovarian granulosa-like tumor cell line, were obtained from Guangzhou Cell Cook Biotech Co., Ltd. The cells were cultured in Dulbecco’s modified Eagle’s medium (DMEM) supplemented with 10% fetal bovine serum (Gibco, Waltham, MA, USA), 100 U/mL penicillin, and 100 µg/mL streptomycin at 37 °C and 5% CO_2_. Ti_3_C_2_ nanosheets was dissolved in DMEM medium at a concentration of 1 mg/mL. KGN cells were respectively treated with 25, 50 and 100 µg/mL Ti_3_C_2_ nanosheets for 24 h, while control group without any treatment. According to the instructions, prepare stock solutions of 3-MA (MCE, China) and Rapamycin (GLPBIO, USA) using distilled water and DMSO, respectively. Referring to the existing reports, 3-MA or Rapamycin were diluted in DMEM containing 2% or 10% FBS to concentrations of 1 mM (3-MA) and 100 nM (Rapamycin), followed by co-incubation with Ti_3_C_2_ nanosheets or not for 24 h [31, 32].

### ELISA analysis

Female mice undergo a 3–5 d hormonally controlled estrous cycle [33]. Serum from the different groups of mice were collected after treatment with Ti_3_C_2_ nanosheets and saline for 3 days. The serum was separated by centrifugation at 3000 rpm for 5 min and then stored at -80 ℃. The serum levels of testosterone (T), follicle-stimulating hormone (FSH), progesterone (P_4_), luteinizing hormone (LH), and estradiol (E_2_) were evaluated via enzyme-linked immunosorbent assay (ELISA) kits and conducted by Shanghai Yanhui Biotechnology Co., Ltd. Detection limit of ELISA kit: E_2_ (0–120 pg/mL), P_4_ (0–16 ng/mL), FSH (0–80 mIU/mL), LH (0–8 mIU/mL), T (0–24 ng/mL).

KGN cells were seeded at a density of 1.2 × 10^6^ cells per well in a 6-well plate and cultured in DMEM supplemented with 10% FBS for 24 h. To mimic the in vivo conditions of human ovarian granulosa cells, 10 µmol/L testosterone (as an androgen substrate for estradiol synthesis in KGN cells) (GLPBIO, USA) [34] and 500 ng/mL FSH (as a stimulant for estradiol secretion in KGN cells) [35, 36] were used. Cultured KGN cells were treated with testosterone, FSH and Ti_3_C_2_ nanosheets (prepared in DMEM supplemented with 2% FBS) for 24 h, while control group only treatment with testosterone and FSH. Subsequently, supernatants from the stimulated KGN cells were collected for the measurement of estradiol and progesterone by Shanghai Chuangxiang Biological Technology Co., Ltd. Detection limit of ELISA kit: E_2_ (15.625–500 pg/mL), P_4_ (0.9375–30 ng/mL).

According to the instructions of the ELISA kit, accuracy: the correlation coefficient (R value) for the standard curve linear regression and expected concentration is greater than or equal to 0.9900. Specificity: no cross-reactivity with other soluble analogs.

### Cell viability assay

The manufacturer’s cell fluorescence counting kit (CCK-F) (C2013S; Beyotime, China) was used to assess the viability of KGN cells. In BeyoGold™ all-black 96-well plates, KGN cells (5 × 10^3^ cells/well) were seeded and allowed to grow overnight before being exposed to Ti_3_C_2_ nanosheets for a full day. The mixture was then incubated for 30 min at 37 °C after 100 µL of calcein AM detection solution was added to each well. A luciferase plate analyzer (Thermo Scientific, USA) was used to directly observe the fluorescence intensity. Finally, the cell viability was determined using the fluorescence intensity.

### Western blot analysis

Total proteins from ovarian tissues or KGN cells were extracted with pre-cooled RIPPA lysis buffer with the addition of PMSF (RIPPA: PMSF = 100: 1) (Beyotime, China). Protein concentration was measured using a BCA protein assay kit following a 30-minute ice-soaked lysate (Beyotime, China). Load 15 µl of ovarian tissues and KGN cells protein samples per well, and protein samples were separated by 6%, 8%, or 12% SDS‒PAGE, transferred to polyvinylidene fluoride (PVDF) membranes pre-activated with methanol, and subsequently blocked with 5-10% skim milk powder for 1 h at 37 °C. Specific primary antibodies were incubated on these PVDF membranes for an entire night at 4 °C. The primary antibodies that were employed as follows: LC3, p-AKT, AKT, ULK1, mTOR, P62/SQSTM1, p-ULK1 (1:1000; CST, USA), p-mTOR (1:1000; Millipore Sigma, USA), ATG5 (1:1000; Gene Universal, USA), PI3K (1:1000; Wanleibio, China), β-actin (1:1,000; ZSGB-BIO, China), CYP19A1 (1:1000; BOSTER, China), FSHR (1:500, LifeSpan Biosciences, China), HSD17β1 and HSD3β2 (1:1000, Proteintech, China) and CYP11A1, StAR (1:2000; Abcam, USA). On the second day, the PVDF membranes were washed four times with PBST before being incubated with horseradish peroxidase-coupled secondary antibody (1:1000; BOSTER, China) at 37 °C for 1 h. Finally, enhanced chemiluminescence (ECL) (NCM Biotech, China) was used to observe the targeted bands, and ImageJ software was used to quantify the band densities.

### Immunofluorescence (IF)

KGN cells were inoculated in 24-well cell climbing slices at a density of approximately 5 × 10^4^ cells per well. Cultured KGN cells were exposed to different concentrations of Ti_3_C_2_ nanosheets, 3-MA or RAPA for 24 h and fixed with 4% paraformaldehyde, then washed once with PBS, and incubated with Triton X-100 (0.1%) at room temperature for 15 min. The cells were then blocked with 5% BSA at 37 °C for an hour after being cleaned with PBS, and incubated with primary antibody against LC3 for the entire night. The next day, goat anti-mouse affinity-purified IgG conjugated with Dylight 488 (Abbkine, Wuhan, China) was used to observe the labeled proteins. Nuclei were stained with DAPI (Beyotime, China) for 10 min, A Nikon confocal microscope (Tokyo, Japan) was used to take pictures of the slides after they had been mounted with an anti-fluorescence quenching solution.

### Statistical analysis

All experiments were repeated independently at least three times. Statistical differences between groups were determined by one-way ANOVA followed by Dunnett’s test or two-way ANOVA followed by Turkey′s test (GraphPad Prism Software 8.0 Inc., San Diego, CA, USA). The results are presented as the means ± standard deviations (SDs). Significant statistical difference is defined as *P* < 0.05. The elements of the graphical abstract are derived from Figdraw.com.

## Results

### Ti_3_C_2_ nanosheets can enter ovarian tissues

**Fig. 1:**
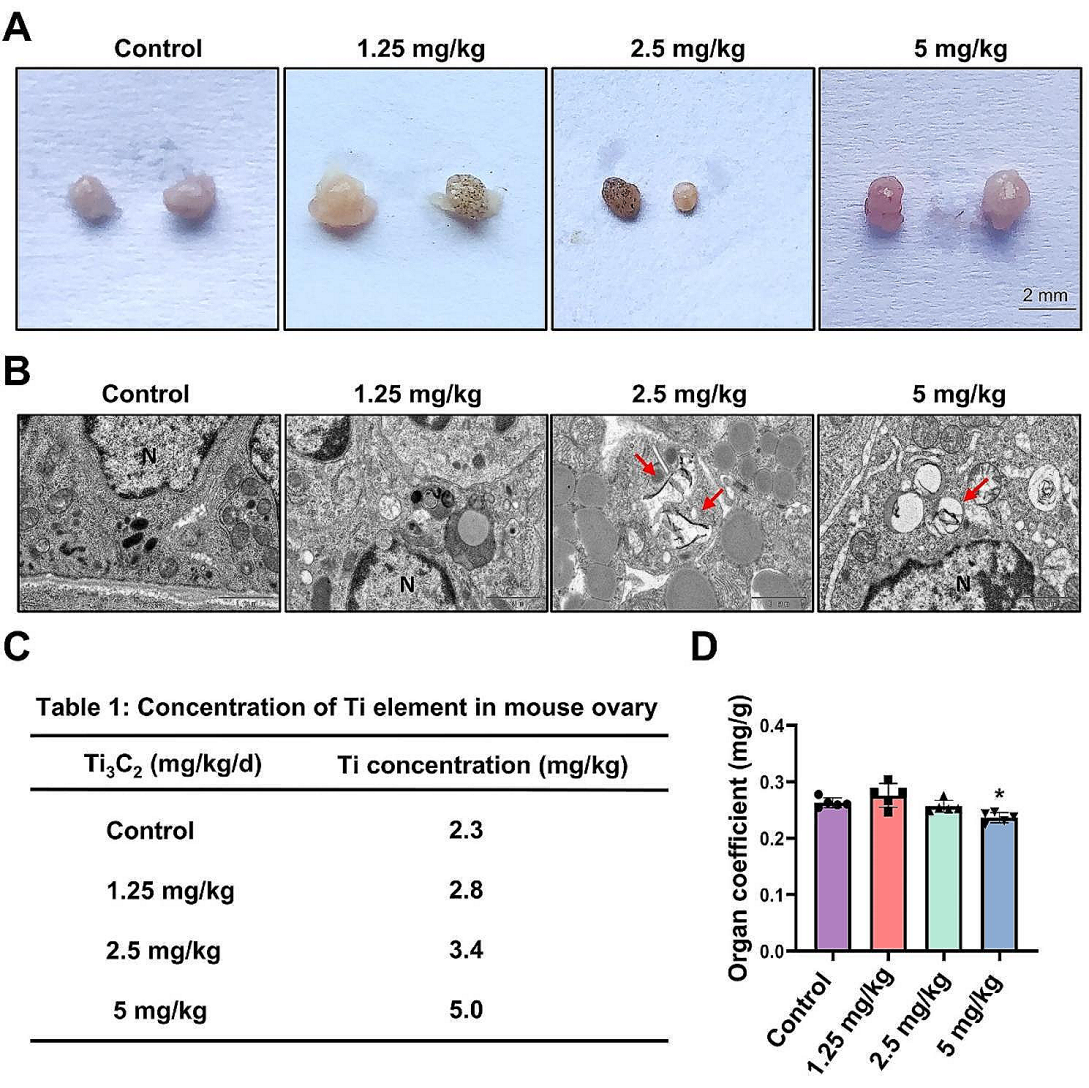
Ti_3_C_2_ nanosheets can enter ovarian tissues. (**A**) Anatomical morphology of mouse ovaries (scale bars = 2 mm). (**B**) The distribution of Ti_3_C_2_ nanosheets in ovarian tissues was observed via TEM (scale bars = 1 μm). Red arrows indicate Ti_3_C_2_ nanosheets. N: nucleus. (**C**) Ti content in ovarian tissues was assessed by ICP‒MS (*n* > 15). (**D**) The organ coefficients of the ovaries at various dosages (*n* = 5 mice per group). All data are presented as the means ± standard deviations (*P < 0.05), compared with the control group

The morphology of Ti_3_C_2_ nanosheets was analyzed by TEM, revealing that the lateral size of the ultra-thin nanosheets was approximately 1–2 μm (Additional file 1: Fig. S1A). Atomic force microscopy (AFM) analysis indicated that the thickness of the nanosheets is around 2–3 nm (Additional file 1: Fig. S1B). The crystalline structure of the as-synthesized sample was characterized by XRD and Raman. The peak of about 6.9^◦^ in sample is the (002) peak of MXene. It can be seen from the Raman spectra that as-prepared samples exhibit two broad peaks at nearly around 412 and 620 cm^− 1^, which were attributed to the energy-gap modes of the in-plane Ti, C, and surface functional groups (Additional file 1: Fig. S1C, D). We performed TEM and ICP‒MS analyses to determine the distribution of Ti_3_C_2_ nanosheets in ovarian tissue. A mouse model was generated by intravenous injection of different doses of Ti_3_C_2_ nanosheets for three days. As shown in Fig. 1A, black substances on the surface of ovarian tissues were observed in the Ti_3_C_2_ nanosheets-exposed group, notably accentuated in the 1.25 mg/kg bw/d and 2.5 mg/kg/ bw/d groups, whereas no such substances were observed in the control group. Interestingly, akin to ovarian tissue, other organs might have been exposed to Ti_3_C_2_ nanosheets, including vital organs like the heart, liver, spleen, lungs, and kidneys. Among the organs we collected, it was observed that the livers and lungs of the exposed group were noticeably darker compared to those of the control group (Additional file 1: Fig. S2). Our TEM analysis showed that Ti_3_C_2_ nanosheets were deposited in the ovarian granulosa cells of mice in the 2.5 mg/kg bw/d and 5 mg/kg bw/d groups (Fig. 1B). Furthermore, Ti content in the ovarian tissue of the Ti_3_C_2_ nanosheets-treated group was higher than that of the control group (Fig. 1C). Compared to the control group, the organ coefficient of mouse ovaries was significantly reduced after exposure to 5 mg/kg bw/d Ti_3_C_2_ nanosheets (Fig. 1D) (*P* < 0.05). These findings provide confirmation that following intravenous injection of Ti_3_C_2_ nanosheets in mice, the nanosheets can enter ovarian tissue and deposit in granulosa cells, thereby reducing the ovarian organ coefficient.

### **Exposure to Ti**_**3**_**C**_**2**_**nanosheets leads to follicular development derailment and an imbalance in ovarian hormone secretion**

**Fig. 2:**
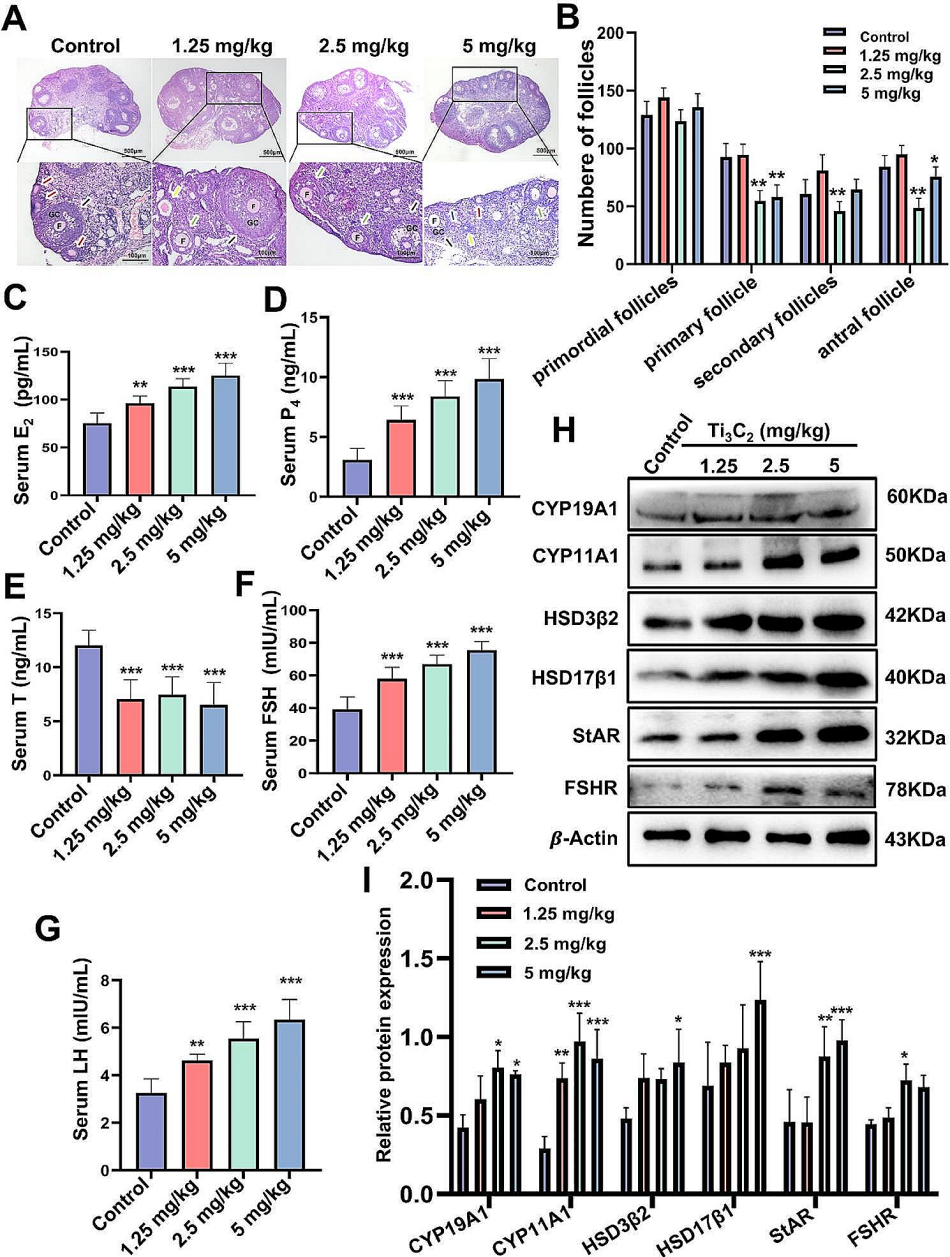
Effects of Ti_3_C_2_ nanosheets on follicular development and hormone secretion in mouse ovaries. (**A**) HE-stained sections displaying the histology of the ovaries (scale bar = 500 and 100 μm). Primordial follicles are shown by the blue arrows, primary follicles by the red arrows, secondary follicles by the green arrows, antral follicles by the black arrows, and atretic follicles by the yellow arrows. GC: granular cell; F: follicle. (**B**) Statistics of the number of follicles (*n* = 3 mice per group). The serum hormone levels of E_2_ (**C**), P_4_ (**D**), T (**E**), FSH (**F**) and LH (**G**) were determined by ELISA (*n* = 9 mice per group). (**H**) Western blotting was used to analyze the protein levels of CYP19A1, CYP11A1, HSD3β2, HSD17β1, StAR and FSHR in ovarian tissues. (**I**) Corresponding quantitative data of related protein levels (*n* = 3 independent repetitions). All data are presented as the means ± standard deviations (*P < 0.05, **P < 0.01, ***P < 0.001), compared with the control group

To further verify the impact of Ti_3_C_2_ nanosheet exposure on the ovary, we investigated ovarian functions, including follicular development and hormone secretion, after intravenous injection of Ti_3_C_2_ nanosheets for three days. HE staining was employed to observe the histological morphology of the ovary and the number of follicles in each stage was counted. As shown in Fig. 2A, B, the number of primary and antral follicles was considerably lower in the 5 mg/kg bw/d group compared to the control group. Furthermore, there were fewer primary, secondary, and antral follicles in the 2.5 mg/kg bw/d group as compared to the control group. There was no difference observed in the number of primordial follicles in each group. Cholesterol typically exists in cells in the form of lipid droplets, which are small vesicles utilized for storing and regulating lipids. Since cholesterol serves as a precursor for steroid hormone synthesis, the increase in lipid droplets may be associated with an increase in hormone synthesis. TEM results show that compared to the control group, there is a significant increase in lipid droplets in ovarian tissue after treatment with Ti_3_C_2_ nanosheets (Additional file 1: Fig. S4A). We used ELISA to measure the levels of serum sex hormones, including E_2_, P_4_, T, FSH, and LH. As shown in Fig. 2C, D, F, G, compared to the control group, exposure to Ti_3_C_2_ significantly increased the levels of E_2_, P_4_, FSH, and LH. However, the serum T levels decreased in all the treatment groups (Fig. 2E). To determine whether Ti_3_C_2_ nanosheets interfere with the expression of enzymes involved in hormone synthesis, we measured the protein levels of relevant steroid hormones enzymes, including CYP11A1, CYP19A1, StAR, HSD17β1, HSD3β2 and FSHR. Western blotting analysis revealed that treated with 5 mg/kg bw/d of Ti_3_C_2_ nanosheets markedly increased the protein levels of CYP11A1, CYP19A1, StAR, HSD17β1, HSD3β2 and treated with 2.5 mg/kg bw/d, there was a significantly increase in the protein level of FSHR (Fig. 2H, I). Taken together, these findings suggest that exposure to Ti_3_C_2_ nanosheets can lead to changes in follicle morphology and increased production of steroid hormones enzymes, resulting in increased secretion of E_2_, P_4_, FSH, and LH and decreased secretion of T, leading to hormonal imbalance.

### **Exposure to Ti**_**3**_**C**_**2**_**nanosheets impairs autophagic flux and inhibits the PI3K/AKT/mTOR signaling pathway in ovarian tissues**

**Fig. 3:**
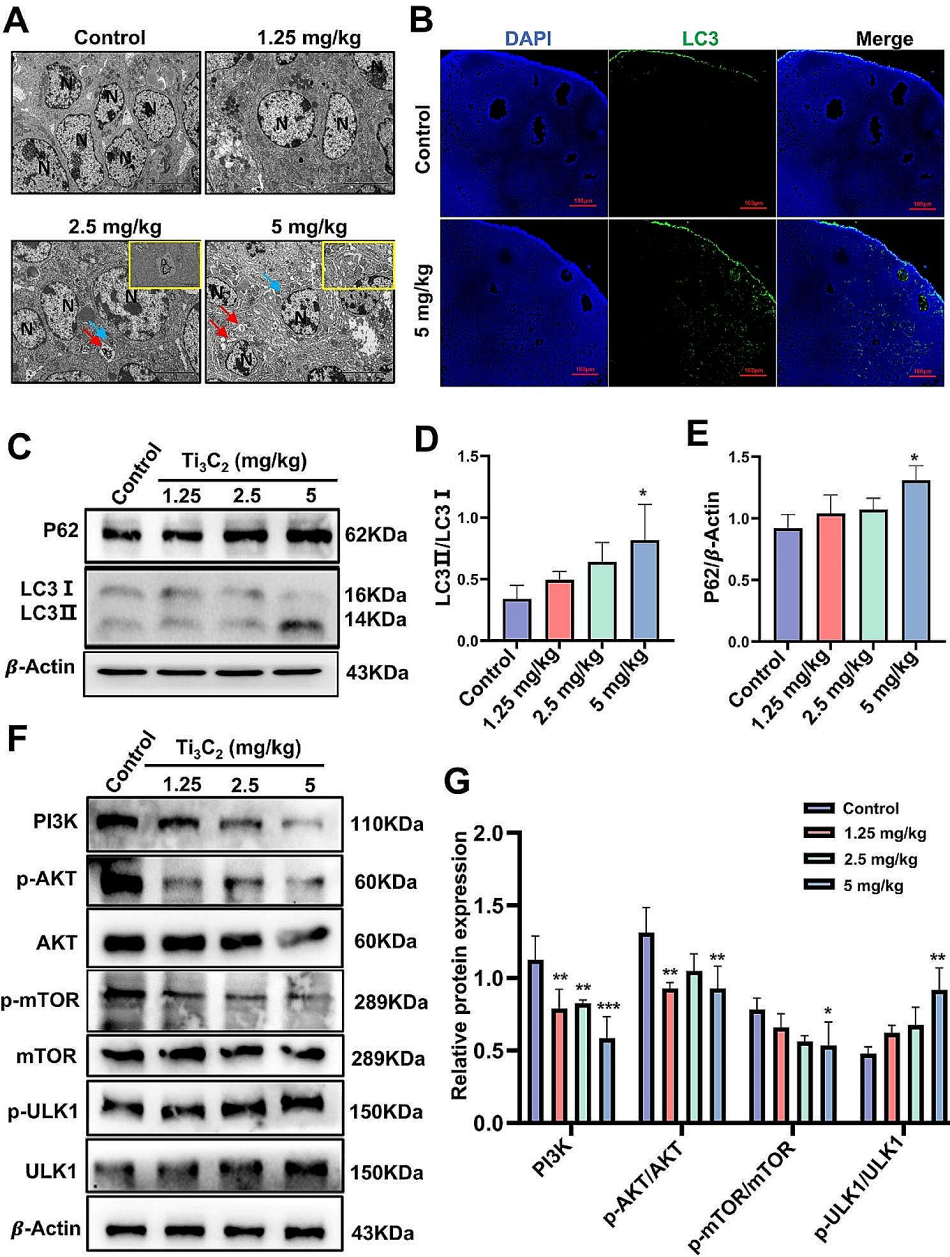
Ti_3_C_2_ nanosheets impaired autophagic flux and inhibited the PI3K/AKT/mTOR signaling pathway in mouse ovary tissue. **A** Autophagy in ovarian tissues were observed via TEM. The blue arrow represents autophagosomes, which have a double membrane structure, while the red arrow represents autolysosomes, which have a single-layer membrane structure and contain degraded organelles. N: nucleus (scale bars = 5 μm and 1 μm). **B** Immunofluorescence was used to measure the expression of LC3 in the ovarian granulosa cells of mice. The DAPI represents nucleus. The LC3 represents autophagy (scale bar = 100 μm). **C, F** Western blotting was used to evaluate the protein levels of P62, PI3K, p-AKT, AKT, mTOR, p-mTOR, p-ULK1 and ULK1 and the LC3II/I ratio in ovarian tissues. **D**, **E**, **G** Quantitative data that correlates with relevant protein levels (*n* = 3 independent repetitions). All data are presented as the means ± standard deviations (*P < 0.05, **P < 0.01, ***P < 0.001), compared with the control group

Female mice undergo a 3- to 5-d, hormonally controlled estrous cycle. The mice were exposed to different doses (1.25, 2.5, and 5 mg/kg bw/d) of Ti_3_C_2_ nanosheets for three days. By the fourth day, all the mice might have returned to estrus, exhibiting an increase in the quantity of antral follicles and atretic follicles. During the natural estrous cycle in mice, the increase in atretic follicles is triggered by various cell death pathways, among which autophagy is one of the cell death mechanisms. Autophagy dysfunction is considered an emerging mechanism of nanomaterial toxicity. To determine whether the imbalance of hormone secretion induced by Ti_3_C_2_ nanosheets may be related to accompanying dysfunction of autophagic flux. TEM results revealed the presence of autophagosomes and autolysosomes in the ovaries of the 2.5 and 5 mg/kg bw/d groups, whereas they were barely observed in the control and 1.25 mg/kg bw/d group (Fig. 3A). Light chain 3 (LC3) is a microtubule-associated protein. LC3 (LC3-I) conjugates with phosphatidylethanolamine to form the LC3-phosphatidylethanolamine conjugate (LC3-II), which is attracted to the autophagosome membrane and is thought to be a useful indicator of autophagy. The immunofluorescence results of LC3, which indicated the accumulation of autophagosomes, were consistent with the TEM results and primarily expressed in the granulosa cells (Fig. 3B). SQSTM1, also known as the autophagy receptor sequestosome 1 (SQSTM1, P62), another important indicator of autophagy degradation, which physically connects autophagy cargo to the autophagosome membrane and represents autophagic flux. Importantly, a significant increase in the LC3II/I ratio and P62 protein levels were apparent in the 5 mg/kg bw/d groups, indicating autophagy activation and impaired autophagic flux in mouse ovary tissues (Fig. 3C-E). The PI3K/AKT/mTOR signaling pathway, as one of the upstream pathways of autophagy, plays a crucial role in regulating the process of cellular autophagy. ULK1 is a key initiation factor in the autophagy process. When the PI3K/AKT/mTOR signaling pathway is inhibited, ULK1 is activated, promoting the progression of autophagy. Therefore, we examined the protein expression levels of PI3K, p-AKT, AKT, p-mTOR, mTOR, p-ULK1 and ULK1 by Western blotting. It was found that compared to the control group, the proteins levels of PI3K, p-AKT/AKT, and p-mTOR/mTOR decreased, while the proteins levels of p-ULK1/ULK1 increased in the ovarian tissue of the 5 mg/kg bw/d group (Fig. 3F, G). The results indicated that the PI3K/AKT/mTOR signaling pathway was inhibited, while ULK1 was activated. All the above results collectively demonstrate that exposure to Ti_3_C_2_ nanosheets can activate autophagy through the PI3K/AKT/mTOR signaling pathway in ovarian granulosa cells. Therefore, we speculate that the decrease in antral follicles and increase in atretic follicles in the ovaries induced by Ti_3_C_2_ nanosheets may be due to autophagy activation and blockade of autophagic flux.

### Intracellular distribution of Ti_3_C_2_ nanosheets and increases in estradiol and progesterone biosynthesis in KGN cells

**Fig. 4:**
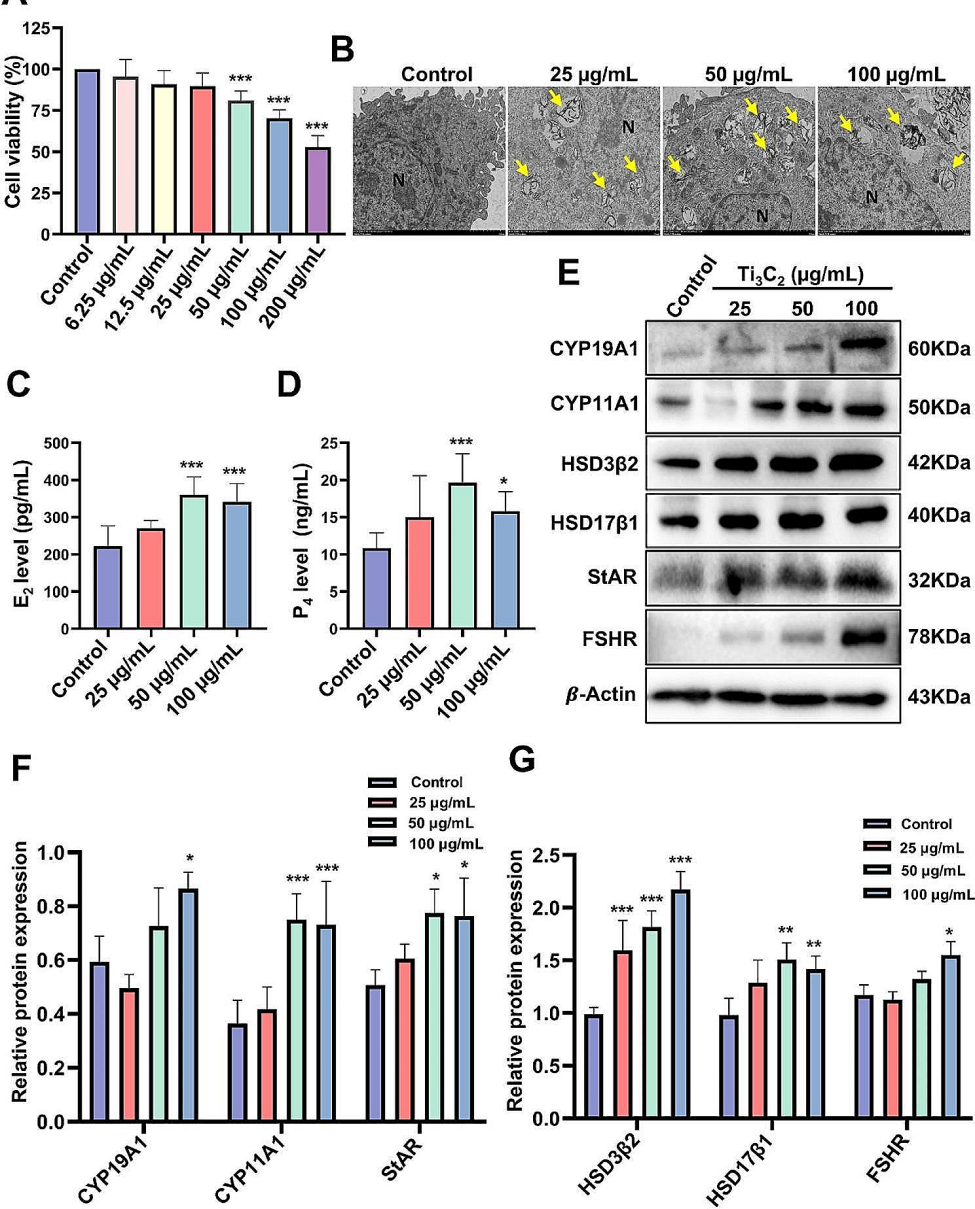
Effect of Ti_3_C_2_ nanosheet exposure on the viability and hormone secretion of KGN cells. **A** CCK-F test was used to assess the viability of KGN cells (*n* = 3 independent repetitions). **B** The cellular ultrastructure of KGN cells were examined by TEM (scale bar = 2 μm). Yellow arrows displayed Ti_3_C_2_ nanosheets. N: nucleus. **C**, **D** The levels of estradiol and progesterone in the cell culture supernatant (*n* = 3 independent repetitions). **E** Western blotting was used to analyze the protein levels of CYP19A1, CYP11A1, HSD3β2, StAR, HSD17β1 and FSHR. **F**, **G** Corresponding quantitative data of related protein levels (*n* = 3 independent repetitions). The detection of steroid synthesis enzymes and hormone secretion requires treatment with testosterone (10 µmol/L) and FSH (500 ng/mL) for 24 h. All data are presented as the means ± standard deviations (*P < 0.05, **P < 0.01, ***P < 0.001), compared with the control group

In the ovary, there are two main types of steroidogenic cells. The theca cells (along with a small number of stromal cells) produce androgens, while granulosa cells are responsible for converting androgens into estradiol [37]. Therefore, we chose the ovarian granulosa cell line (KGN cells) for validation in vitro. TEM analysis was applied to confirm the nanosheets distribution and ultrastructural alterations in KGN cells treated with different doses of Ti_3_C_2_ nanosheets. Imaging revealed clear evidence of nanosheet internalization by the treated cells. Specifically, the majority of Ti_3_C_2_ nanosheets were found to be localized within the cytoplasm of KGN cells (Fig. 4B). Cell viability was analyzed by calcein AM cell activity assay (CCK-F) and was found to be significantly reduce in the 50 µg/mL and 100 µg/mL Ti_3_C_2_ nanosheet treatment groups (Fig. 4A). A live/dead cell assay was employed to differentiate live and dead cells using a two-color fluorescent dye, and the number of dead cells (denoted by PI staining) did not change significantly after Ti_3_C_2_ nanosheets exposure (Additional file 1: Fig. S3). According to the results of the CCK-F assay, we chose 25, 50, and 100 µg/mL as the concentrations of Ti_3_C_2_ for subsequent experiments. To investigate the effects of Ti_3_C_2_ nanosheets on hormone biosynthesis in KGN cells, we simulated the in vivo conditions of estradiol synthesis in human ovarian granulosa cells. We used 10 µmol/L testosterone as the substrate and added 500 ng/mL FSH as a stimulant to promote estradiol secretion from granulosa cells. The precursor molecule stored in lipid droplets (LDS), cholesterol, is the precursor for progesterone synthesis. Cholesterol can be converted into progesterone through a series of enzymes, including cholesterol side-chain cleavage enzyme (CYP11A1), 3β-hydroxysteroid dehydrogenase (HSD3β2), and progesterone synthase. Compared to the control group, TEM revealed a significant increase in lipid droplets in the 50 µg/mL and 100 µg/mL group (Additional file 1: Fig. S4B). We collected the cell culture supernatant and measured the levels of estradiol and progesterone. The results showed that compared with control group, the level of estradiol and progesterone significantly increased in the 50 µg/mL and 100 µg/mL groups (Fig. 4C, D). In addition, we also evaluated the protein levels of the synthetic enzymes associated with the production of estradiol and progesterone. Compared to the control group, the protein levels of CYP11A1, StAR, HSD17β1, and HSD3β2 significantly increased in the 50 µg/mL and 100 µg/mL groups, while the protein levels of CYP19A1 and FSHR only significantly increased in the 100 µg/mL group (Fig. 4E-G). The above results indicate that Ti_3_C_2_ nanosheets were internalized by the KGN cells, had deposited in the cytoplasm, and promoted the expression of key enzymes in steroid synthesis, leading to a significant increase secretion of steroid hormones (E_2_ and P_4_).

### **Exposure to Ti**_**3**_**C**_**2**_**nanosheets impairs autophagic flux and inhibits the PI3K/AKT/mTOR signaling pathway in KGN cells**

**Fig. 5:**
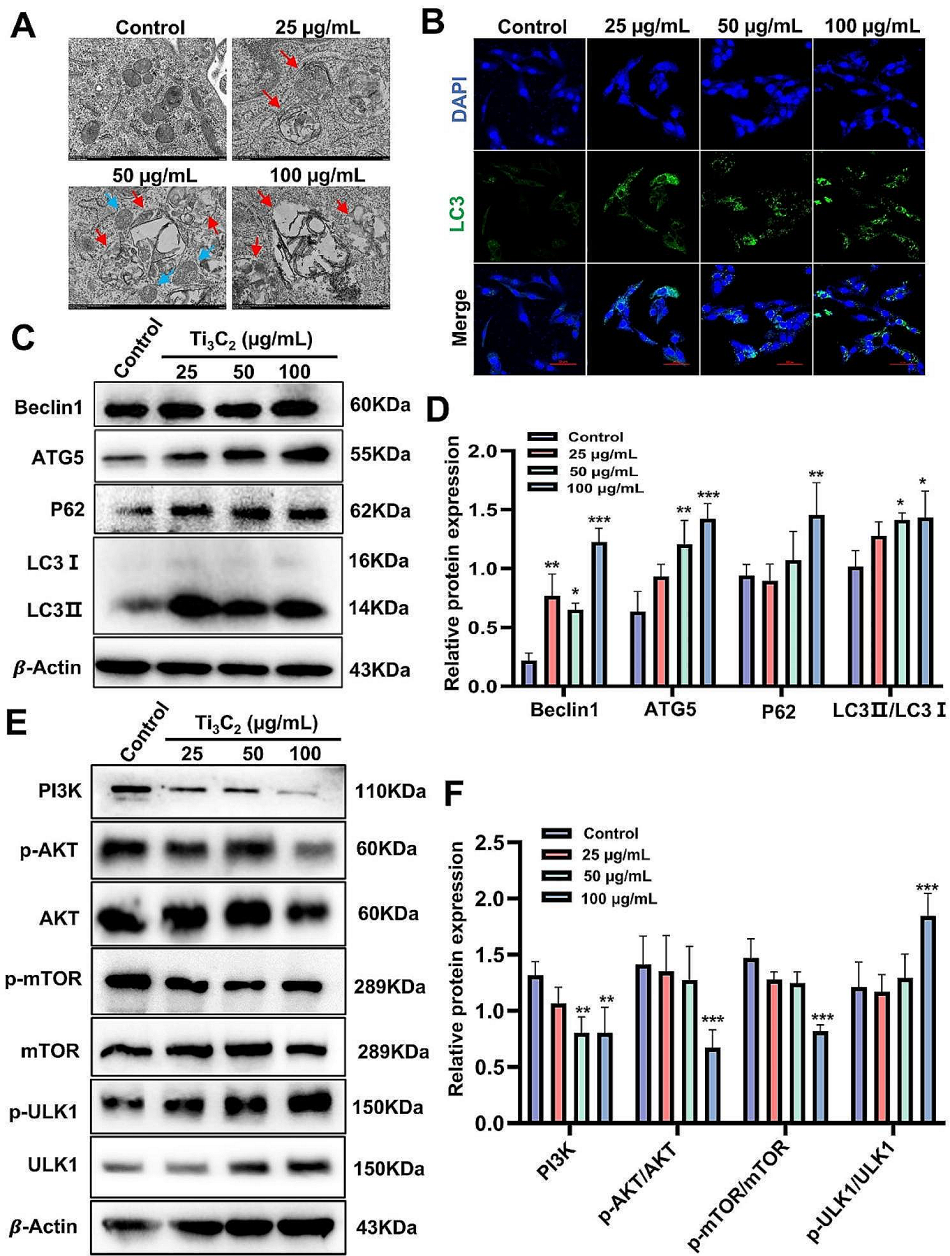
Exposure to Ti_3_C_2_ nanosheets impairs autophagic flux and inhibits the PI3K/AKT/mTOR signaling pathway in KGN cells. **A** Autophagosomes in KGN cells were evaluated via TEM. The blue arrow represents autophagosomes, which have a double membrane structure, while the red arrow represents autolysosomes, which have a single-layer membrane structure and contain degraded organelles (scale bar = 500 nm). **B** Images of LC3 (green) identified by immunofluorescence (scale bar = 50 μm). **C, E** Western blotting was used to analyze the protein levels of Beclin-1, ATG5, P62, the LC3II/I ratio, PI3K, p-AKT, AKT, mTOR, p-mTOR, p-ULK1 and ULK1 in KGN cells. **D, F** Quantitative data that correlates with relevant protein levels (*n* = 3 independent repetitions). All data are presented as the means ± standard deviations (*P < 0.05, **P < 0.01, ***P < 0.001), compared with the control group

TEM revealed a significant increase in the number of autophagosomes and autolysosomes in Ti_3_C_2_ nanosheet-treated KGN cells compared to the control group (Fig. 5A). The autophagosome marker LC3 was used to measure autophagy activity. Confocal microscopy revealed that the number of LC3-positive puncta was significantly augment in KGN cells after treatment with Ti_3_C_2_ nanosheets for 24 h (Fig. 5B). Western blotting analysis showed that compared to the control group, exposure to Ti_3_C_2_ nanosheets at a concentration of 100 µg/mL led to a significant increase in P62 and the LC3II/I ratio. The elevated protein levels of P62 suggest that Ti_3_C_2_ nanosheets exposure significantly blocked autophagic flux. Beclin1 belongs to the family of autophagy-related protein family and plays a role in regulating the formation of autophagosomes, promoting their maturation. ATG5 is an essential part of autophagy that promotes the lipidation of LC3-I to LC3-II. Western blotting analysis revealed an increase in Beclin1 and ATG5 protein levels after Ti_3_C_2_ nanosheets treatment (Fig. 5C, D). These results indicate that treatment with Ti_3_C_2_ nanosheets can promote the formation of autophagosomes and block autophagic flux. To further elucidate the upstream regulatory factors of autophagy induced by Ti_3_C_2_ nanosheets, we evaluated protein level of the PI3K/AKT/mTOR signaling pathway by Western blotting. As shown in Fig. 5E, F, significant decreases in the PI3K, p-AKT/AKT and p-mTOR/mTOR ratios were observed in the 100 µg/mL group, while the p-ULK1/ULK1 ratio was significantly increased. These findings suggest that the activation of autophagy in KGN cells following exposure to Ti_3_C_2_ nanosheets could be attributed to the PI3K/AKT/mTOR signaling pathway.

### **Autophagy initiation inhibits by 3‑MA partially alleviates the Ti**_**3**_**C**_**2**_**‑induced imbalance in hormone secretion in KGN cells**

**Fig. 6:**
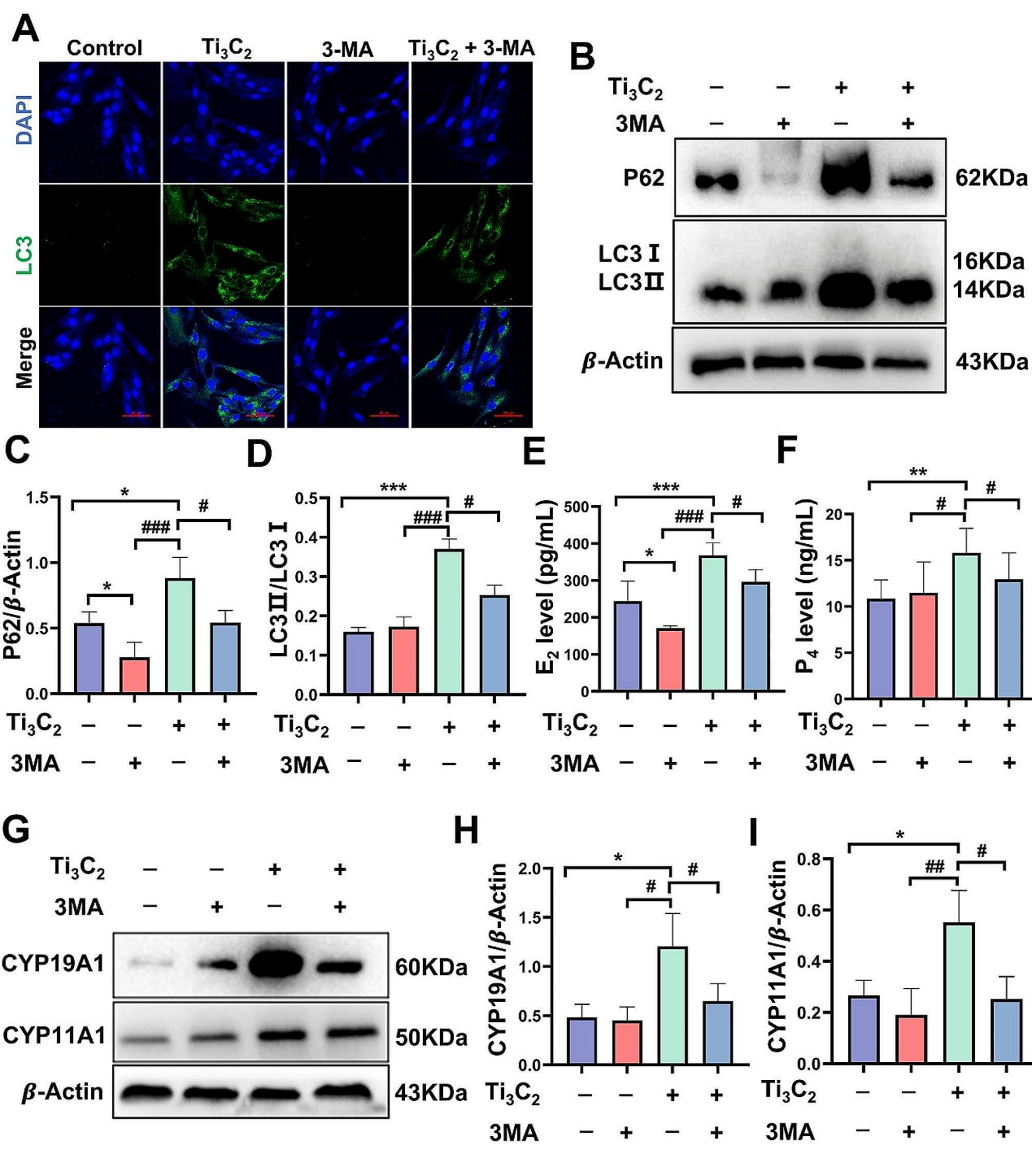
The inhibition of autophagy initiation by 3-MA alleviated the imbalance of hormone secretion in Ti_3_C_2_-induced KGN cells. **A** Immunofluorescence image showing the LC3 fluorescent puncta formed by 3-MA combined with Ti_3_C_2_ nanosheets after 24 h in KGN cells (scale bar = 50 μm). **B**, **G** The protein expression levels of P62, LC3II/I, CYP19A1 and CYP11A1 in KGN cells were determined by Western blotting. **C**, **D**, **H**, **I** Quantitative data that correlates with relevant protein levels (*n* = 3 independent repetitions). **E**, **F** The levels of progesterone and estradiol in the cell culture supernatant (*n* = 3 independent repetitions). The detection of steroid synthesis enzymes and hormone secretion requires treatment with testosterone (10 µmol/L) and FSH (500 ng/mL) for 24 h. All data are presented as the means ± standard deviations (*P < 0.05, **P < 0.01, ***P < 0.001), compared with the control group. (**#***P* < 0.05, **##***P* < 0.01, **###***P* < 0.001), compared with the Ti_3_C_2_ nanosheets-treated group

To further investigate whether the activation of autophagy is indeed associated with the disruption of hormone biosynthesis induced by Ti_3_C_2_ nanosheets, 3-MA was used to inhibit autophagosome formation. KGN cells were treated with 100 µg/mL Ti_3_C_2_ nanosheets either in conjunction with or apart from 1 mM 3-MA. Fluorescence analysis revealed that when Ti_3_C_2_ nanosheets and 3-MA were applied to KGN cells, the amount of green fluorescent LC3 puncta was much lower than in the cells treated with Ti_3_C_2_ nanosheets alone (Fig. 6A). Consistently, the protein levels of P62 and the LC3II/I ratio were significantly reduced after cotreatment with Ti_3_C_2_ nanosheets and 3-MA for 24 h (Fig. 6B, C, D). However, the lower expression level of P62 suggesting that Ti_3_C_2_ nanosheets treatment with 3-MA boosted autophagic flux, thereby alleviating the inhibition of cellular degradation caused by autophagy. We noticed that compared to the Ti_3_C_2_ nanosheets group, after autophagy inhibition, the levels of progesterone and estradiol in the culture medium were partially reduced (Fig. 6E, F). Western blotting was used to determine the protein expression levels of the synthetic enzymes CYP11A1 and CYP19A1 in KGN cells. Cotreatment with 3-MA alleviated the increase of cell hormone enzymes triggered by Ti_3_C_2_ (Fig. 6G, H, I). These results indicate that inhibiting autophagy can restore the excessive increase in hormone secretion capabilities of KGN cells induced by exposure to Ti_3_C_2_ nanosheets, and this shows that Ti_3_C_2_ nanosheet exposure may cause cellular malfunction by activating autophagy and blocking autophagic flux.

### Blocking autophagic flux with rapamycin aggravates the imbalance in hormone secretion in Ti_3_C_2_‑treated KGN cells

**Fig. 7:**
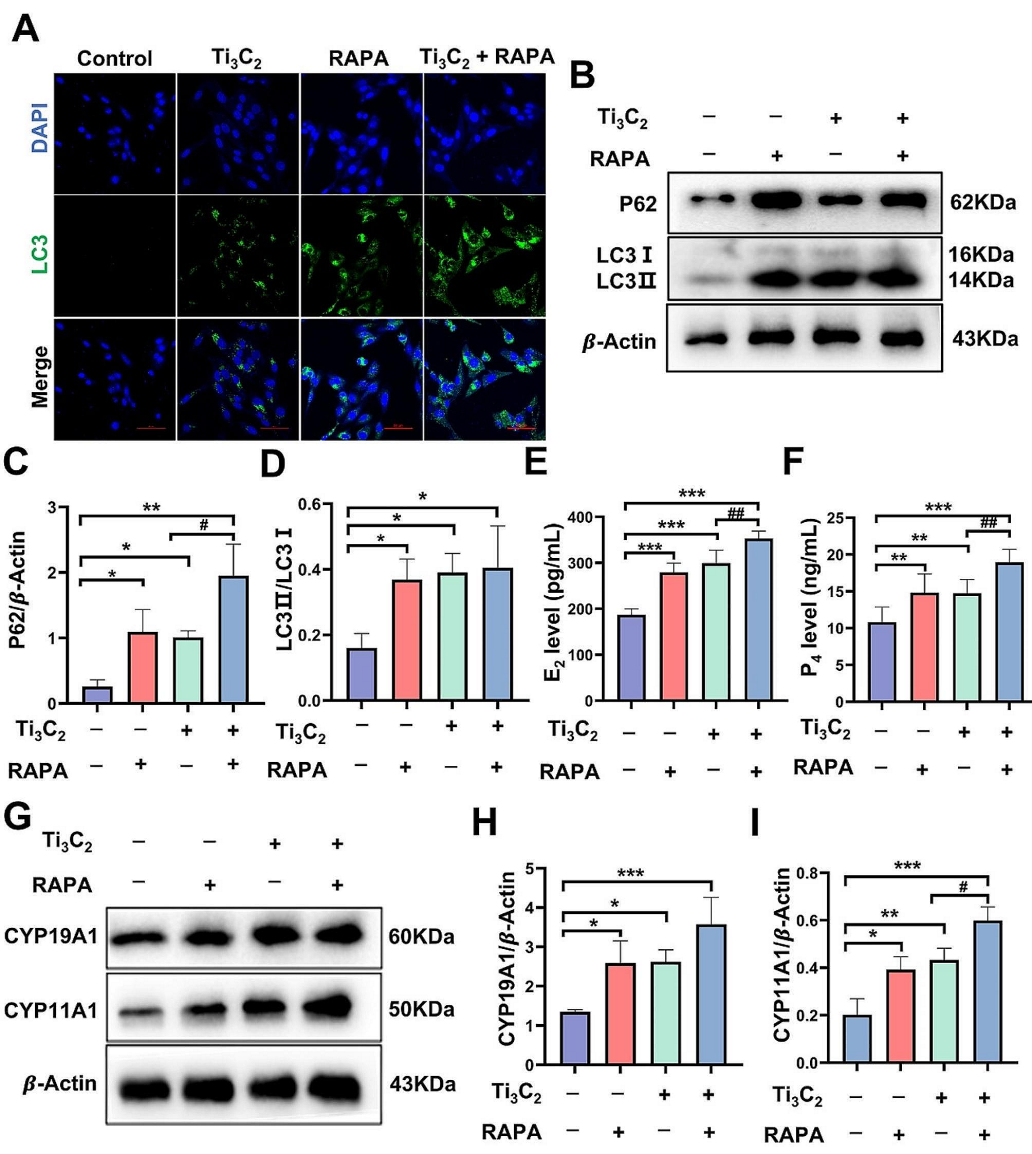
Blocking autophagic flux with rapamycin aggravates the Ti_3_C_2_‑induced imbalance in hormone secretion in KGN cells. **A** Immunofluorescence image showing the LC3 fluorescent puncta formed by RAPA combined with Ti_3_C_2_ nanosheets after 24 h in KGN cells (scale bar = 50 μm). **B**, **G** The protein levels of P62, CYP19A1 and CYP11A1 and the LC3II/I ratio in KGN cells were measured by Western blotting. **C**, **D**, **H, I** Quantitative data that correlates with relevant protein levels (*n* = 3 independent repetitions). **E**, **F** The levels of estradiol and progesterone in the cell culture supernatant (*n* = 3 independent repetitions). The detection of steroid synthesis enzymes and hormone secretion requires treatment with testosterone (10 µmol/L) and FSH (500 ng/mL) for 24 h. All data are presented as the means ± standard deviations (*P < 0.05, **P < 0.01, ***P < 0.001), compared with the control group. (**#***P* < 0.05, **##***P* < 0.01), compared with the Ti_3_C_2_ nanosheets-treated group

Finally, rapamycin (RAPA) is a specific inhibitor of the mTOR protein and an activator of autophagy. Therefore, we investigated the impact of RAPA-induced activation of autophagy and further blockade autophagic flux on cellular hormone secretion by Ti_3_C_2_-treated KGN cells. KGN cells were treated with 100 µg/mL Ti_3_C_2_ nanosheets with or without the 100 nM RAPA. After treatment with Ti_3_C_2_ nanosheets and RAPA, the green fluorescence of LC3 was significantly enhanced in comparison to that in the Ti_3_C_2_ nanosheet group (Fig. 7A). Compared to treatment with Ti_3_C_2_ nanosheets alone, cotreatment with Ti_3_C_2_ nanosheets and RAPA induced a higher protein level of P62 (Fig. 7B, C). These results indicate that RAPA partially aggravates the Ti_3_C_2_-induced impairment of autophagic flux and further enhances autophagy levels. We observed that compared with a single treatment of Ti_3_C_2_ nanosheets group, after cotreatment with RAPA and Ti_3_C_2_ nanosheets, the levels of estradiol and progesterone in the supernatants of the culture media were increased (Fig. 7E, F). Moreover, the Western blotting analysis results showed that, compared with that in the Ti_3_C_2_ nanosheets treatment group, the combination of RAPA with Ti_3_C_2_ nanosheets treatment increased the protein levels of CYP11A1 in KGN cells (Fig. 7G, I). These results indicated that further blocking autophagic flux with RAPA aggravated the biosynthetic capacity of the hormone enzymes. According to these data, we can conclude that the exacerbated hormone secretion abnormalities in KGN cells caused by Ti_3_C_2_ nanosheets may be associated with the accompanying activation of autophagy and blockade of autophagic flux.

## Discussion

MXenes are a new type of emerging nanomaterial with enormous potential for applications in nanomedicine. Currently, titanium carbide Ti_3_C_2_Tx nanomaterials are the most mature and have received considerable attention in areas such as antibacterial applications, drug delivery, treatment of allograft vascular lesions, and inflammatory diseases due to their unique physical and material properties [38–40]. However, the effects of Ti_3_C_2_ on mammalian reproduction have not been thoroughly studied, which restricts further development of its biomedical applications. In our previous study, we discovered that exposure to Ti_3_C_2_ nanosheets during pregnancy can result in their accumulation in the uterus and placenta, leading to notable neurotoxic effects on offspring [23]. Additionally, Ti_3_C_2_ nanosheets can cause severe impairment of the spermatogenic function of male mice [25]. Therefore, additional in-depth studies and evaluations are needed to assess the safety and potential risks of Ti_3_C_2_ nanosheets in the field of reproduction.

To our knowledge, this is the first study to investigate the effects of Ti_3_C_2_ nanosheets on ovarian function both in vivo and in vitro. Studies have shown that Titanium has a certain expression background in various organs and Ti_3_C_2_ nanosheets were rapidly accumulated in the lungs, liver, and spleen after entering circulation [41], which was consistent with the organ exposure observed by us. Titanium, as a transition metal, exists in the human body, not exceeding 15 mg per 70 kg of body weight. ICP-MS analysis revealed that the titanium content in ovarian tissue exceeds 2 mg/kg. Studies indicated that the Titanium content was also shown to be increased in other organs such as the uterus, placenta, and testes [23, 25]. In our study, significant accumulation of titanium in ovarian tissue appears to have caused a toxic reaction, primarily evidenced by hormonal imbalance and histological changes. According to the results of in vivo experiments in mice in our study, exposure to Ti_3_C_2_ nanosheets can decrease the coefficient of change in ovary weight to BW and have toxic effects on folliculogenesis. Follicles are the basic functional components of the ovary, and the quality and quantity of follicles play crucial roles in the effectiveness of fertilization. Therefore, we counted the number of follicles at different stages and observed reductions in the numbers of primary, secondary, and antral follicles after Ti_3_C_2_ exposure. Additionally, we observed the presence of atretic follicles following Ti_3_C_2_ exposure. We believe that Ti_3_C_2_ exposure disrupts the growth of ovarian follicles, speeds up the depletion of the primordial follicle pool, and interferes with the development of antral follicles. Hormones regulate ovarian follicle development, and the balance of hormones determines whether the developing follicle matures or undergoes atresia [42, 43]. Similarly to ovarian tissue, other endocrine organs such as the brain and pituitary gland may have been exposed to Ti_3_C_2_. FSH and LH are crucial components of the hypothalamic-pituitary-gonadal axis, responsible for regulating the production and fertility of gametes [44]. The secretion of FSH and LH is primarily controlled by hypothalamic GnRH, which binds to membrane receptors on the pituitary gland, activating the adenylate cyclase cAMP protein kinase system to promote the biosynthesis and secretion of FSH and LH [45]. E_2_ and P_4_, the two main hormones secreted by the ovary, regulate the development of the female reproductive system and maintain normal pregnancy process [46, 47]. The levels of estradiol and progesterone are regulated by FSH and LH, and these hormones can also regulate the secretion of FSH and LH from the anterior pituitary gland through negative feedback mechanisms [48]. However, in the process of follicle development, the involvement of androgens as intermediate substrates is indispensable. According to the two-cell, two-gonadotropin hypothesis, LH drives theca cells to produce androgens (androstenedione or testosterone), while granulosa cells convert androgen substrates into estrogen under the influence of FSH [49]. The relationships between these hormones are intricate and mutually regulated. TiO_2_ NPs-induced premature ovarian failure in female mice is associated with abnormal serum parameters [50]. Research has shown that exposure to TiO_2_ NPs leads to a decrease in ovarian weight, inhibits ovarian follicle development, and has been proved closely related to higher levels of FSH and LH, as well as higher levels of activin, follistatin, TGF-β1, and lower level of inhibin-α in mouse [26]. In this study, after treatment with Ti_3_C_2_ nanosheets, the serum levels of E_2_, P_4_, FSH, and LH significantly increased, while the level of T as an intermediate product decreased. It is possible that theca cells were damaged, leading to the inability to produce a certain level of testosterone. Previous studies have shown that mice lacking testosterone and functional androgen receptors will experience accelerated ovarian aging, shortened reproductive lifespan, and infertility [51]. This indicates that the imbalance of sex steroid hormones may be related to the occurrence of follicular atresia.

In the ovaries of females, ovarian steroids are produced by granulosa cells and theca cells of follicles and involve multiple steroidogenic enzymes. Cholesterol is the precursor of all ovarian steroid hormones. It is transported from the outer mitochondrial membrane to the inner mitochondrial membrane through the StAR protein, and then catalyzed by the enzymes HSD3β1 and CYP11A1 within the mitochondria to form pregnenolone. Subsequently, within the smooth endoplasmic reticulum, the other two steroidogenic enzymes, HSD3β2 and CYP17A1 convert pregnenolone into progesterone and androstenedione through consecutive reactions. Lastly, the HSD17β1 and CYP19A1 transports androstenedione to granulosa cells where it is aromatized into estradiol [52–54]. In the present study, we discovered that after being exposed to the Ti_3_C_2_ nanosheets, E_2_ and P_4_ increased, and the trends of key enzymes in the body were the same. In ovarian granulosa cells, FSH binding to FSHR causes the fast activation of numerous signaling molecules. This, in turn, activates the transcription of the CYP19A1 gene and ultimately affects the E_2_ level [55]. Studies found that exposure to TiO_2_ NPs led to increased expression of CYP17A1 in the ovaries, indicating that TiO_2_ may be the cause of the increase in estradiol biosynthesis [56]. Exposure to low levels (5 µg/mL) of ZnO NPs increases aromatase levels, leading to elevated levels of estradiol and reduced levels of estrogen receptor alpha (Esr1) [57]. The production of steroid hormones involves the regulation of the precursor molecule cholesterol stored in lipid droplets (LDs), a process dependent on autophagy mechanisms. Autophagy regulates steroid production by influencing cholesterol transport. Research have demonstrated that reduced autophagy impairs steroid synthesis by decreasing the mobilization of cholesterol substrates stored in LDL [58]. After treating with Ti_3_C_2_ nanosheets, our study found an increasing trend of lipid droplets in ovarian tissues and KGN cells, along with an increased expression of autophagy-related markers. We speculate that the increase in steroid hormone secretion may be closely associated with cholesterol accumulation and enhanced autophagy.

The increase in atretic follicles during the natural estrous cycle of mice is triggered by various cell death pathways, including autophagy and apoptosis. Although autophagy-related genes can be detected throughout the entire ovary, autophagosomes are more commonly observed in the granulosa layer of atretic follicles [59]. Excessive autophagy-induced cell death can alter the quality and quantity of oocytes [60]. For instance, SNP activates autophagy, leading to autophagic flux blockade, increasing apoptosis of ovarian granulosa cells, ultimately resulting in follicular atresia [15]. ZnO-NPs inhibit fertility by activating autophagy, apoptosis, and oxidative stress in developing oocytes of female zebrafish [14]. Autophagy is an important intracellular metabolic process in which cells wrap and form autophagosomes to package cell components or damaged organelles and transport them to lysosomes for degradation. This process involves multiple steps, including autophagosome initiation, formation, maturation, and degradation, also known as autophagic flux. Autophagy is a crucial process that plays a protective role in maintaining cellular homeostasis and normal functioning. However, excessive activation of autophagy and blockade of autophagic flux can lead to cell dysfunction. Beclin1 forms a complex with class III phosphatidylinositol 3-kinase (PI3K) and participates in the initiation and vesicle formation of autophagy [61]. ATG5, a key component of autophagy, regulates the formation of autophagosomes. ATG5 binds to ATG12 and forms a complex with ATG16L1, creating an E3-like ligase complex that promotes the formation and elongation of autophagosomes [62]. LC3 is a hallmark of autophagy that facilitates cargo delivery and the formation and maturation of autophagosomes. Our study demonstrated that Ti_3_C_2_ nanosheets increased the levels of Beclin-1 and ATG5 and promoted the conversion of LC3-I to LC3-II. P62, a monitoring indicator of autophagic flux, increased with high-dose exposure to Ti_3_C_2_ nanosheets, indicating that ovarian tissues and KGN cell exposure to Ti_3_C_2_ nanosheets not only activates autophagy but also inhibits the degradation of autophagosomes, thereby blocking autophagic flux.

Many signaling pathways are involved in regulating autophagy, and mechanistic target of rapamycin (mTOR) serves as a core hub regulating autophagy. These pathways are regulated by different upstream signaling pathways [63]. The PI3K (phosphoinositide-3-kinase)/AKT (protein kinase B)/mTOR (mammalian target of rapamycin) signaling pathway is an important pathway that regulates autophagy. Specifically, Maroua Jalouli et al. reported that allethrin may impair autophagy-related cell apoptosis and induce excessive oxidative stress, most likely via inhibiting the PI3K/AKT/mTOR signaling pathway, and these effects may result in ovarian dysfunction and reduced fertility in female offspring [64]. Our previous research revealed that cellular autophagy is activated via the PI3K/AKT/mTOR signaling pathway, eventually leading to functional impairment in HTR-8/SVneo cells [24]. We found that Ti_3_C_2_ nanosheets treatment decreased the protein level of PI3K, p-AKT/ AKT, and p-mTOR/ mTOR. These results imply that the PI3K/AKT/mTOR signaling pathway may participate in the activation of autophagy in ovarian tissues and KGN cells in response to Ti_3_C_2_ nanosheets exposure. To further confirm the effects of Ti_3_C_2_ nanosheets exposure on autophagy activation and the inhibition of autophagic flux in KGN cells, we treated KGN cells with *3*-methyladenine (3-MA) to suppress autophagy initiation and rapamycin (RAPA) to induce autophagy. We observed that the inhibition of autophagy partially reversed the hormone imbalance in KGN cells previously damaged by Ti_3_C_2_ nanosheets exposure. These results and underlying mechanisms provide a good explanation for the reduction in ovarian follicle numbers and disruption of hormone secretion induced by exposure to the Ti_3_C_2_ nanosheets.

It is well known that an increase in intracellular reactive oxygen species can induce autophagy through the PI3K/AKT/mTOR signaling pathway. However, granulosa cell (GC) death induced by oxidative stress is also a common cause of follicular atresia. Gao et al. confirmed that chronic exposure to TiO_2_ NPs can lead to accumulation in the ovary and cause ovarian damage, resulting in hormonal imbalance and oxidative stress in mice, which is consistent with our results [56]. Further investigations, both in vivo and in vitro studies, have shown that Ti_3_C_2_ has the potential to alter hormone levels and ovarian hormone synthesis enzymes through pathways such as impairing autophagy and accumulating reactive oxygen species (ROS). ROS can affect multiple physiological processes ranging from oocyte maturation to fertilization, embryo development, and pregnancy [65]. Therefore, this could also be a factor contributing to abnormal changes in hormone levels and follicular development. Several studies have indicated that in vitro exposure to ZnO NPs can lead to the internalization of nanoparticles within antral follicles, affecting steroidogenesis pathways, resulting in an increase in estradiol levels and inducing oxidative stress in ovarian antral follicles [57]. In the present study, exposure to Ti_3_C_2_ nanosheets resulted in higher levels of ROS in ovarian tissue and KGN cells compared to the control group, as well as lower levels of antioxidant enzyme activity (Supplementary File 1: Fig. S5). This suggests that under oxidative stress conditions, granulosa cell death can be induced. Additionally, studies have reported that excessive autophagy can trigger self-destruction of cells with oxidative damage. Therefore, we believe that oxidative stress may be an important factor leading to granulosa cell death through autophagy during follicular atresia. As the concentration of Ti_3_C_2_ nanosheets increased, a gradual decrease in ROS and MDA levels was observed. Similar increase trends were also found in the activity of antioxidant enzymes. Research suggests that the fluorine groups on the surface of Ti_3_C_2_ nanosheets are the primary cause of ROS formation [41]. However, it is necessary to gather more conclusive evidence from multiple aspects to understand the reasons behind.

## Conclusions

In summary, we discovered that Ti_3_C_2_ nanosheets can enter the ovaries and deposit in the cytoplasm of granulosa cells, leading to abnormal hormone secretion and impaired follicular development. Exposure to Ti_3_C_2_ nanosheets leads to the activation of autophagy and obstruction of autophagic flux in granulosa cells. Autophagy may affect the transport of cholesterol in the ovaries, suggesting that autophagy dysfunction may be an important factor leading to abnormal hormone secretion and follicular atresia. Ti_3_C_2_ nanosheets can induce oxidative stress and activate autophagy excessively through the PI3K/AKT/mTOR signaling pathway. These results provide experimental evidence for assessing the adverse biological effects of exposure to Ti_3_C_2_ nanosheets in mice. Furthermore, this study, for the first time, elucidated the mechanism by which exposure to Ti_3_C_2_ nanosheets affects the function of ovarian granulosa cells from the perspective of autophagy, providing clues for preventing biological adverse reactions in female exposure. It also offers new insights into the toxic mechanisms of MXene in the reproductive system.

### Electronic supplementary material

Below is the link to the electronic supplementary material.

Supplementary Material 1

## Data Availability

No datasets were generated or analysed during the current study.

## Abbreviations

GC: Granulosa cells
KGN: human ovarian granulosa-like tumor cell line
F: follicle
N: nucleus
LC3: Microtubule-associated protein 1 light chain 3 alpha
ATG5: Autophagy-related gene
P62: Sequestosome 1
3-MA: 3-methyladenine
RAPA: rapamycin
PI3K: phosphoinositide-3-kinase
AKT: protein kinase B
mTOR: mammalian target of rapamycin
E_2_: estradiol
P_4_: progesterone
FSH: follicle-stimulating hormone
LH: luteinizing hormone
T: testosterone
PBS: phosphate-buffered saline
PBST: Tris-bufered-saline with tween
PVDF: Polyvinylidene fluoride
BSA: bovine serum albumin
SDS-PAGE: sodium dodecyl sulfate‒polyacrylamide gel electrophoresis
DAPI: 4’,6-diamidino-2-phenylindole
TEM: transmission electron microscope
ICP‒MS: Inductively coupled plasma‒mass spectrometry
PI: Propidium Iodide
HSD3β2: 3β-hydroxysteroid dehydrogenase
CYP17A1: cytochrome P450c17
HSD17β1: 17β-hydroxysteroid dehydrogenase
CYP19A1: enzyme cytochrome P450arom
StAR: steroidogenic acute regulatory protein
µl: Microliter
h: Hour
KDa: Kilodalton
bw: body weight
d: day
