# Supplementary material for: Ti3C2 nanosheet-induced autophagy derails ovarian functions
Source: J Nanobiotechnology. 2024 May 12;22:242. doi: 10.1186/s12951-024-02495-4 (PMC11089700; doi:10.1186/s12951-024-02495-4)
Supplement: Supplementary file 1 — Supplementary Material 1 [file 12951_2024_2495_MOESM1_ESM.docx]

Additional Information

**Ti_3_C_2_ nanosheet-induced autophagy derails ovarian functions**

**
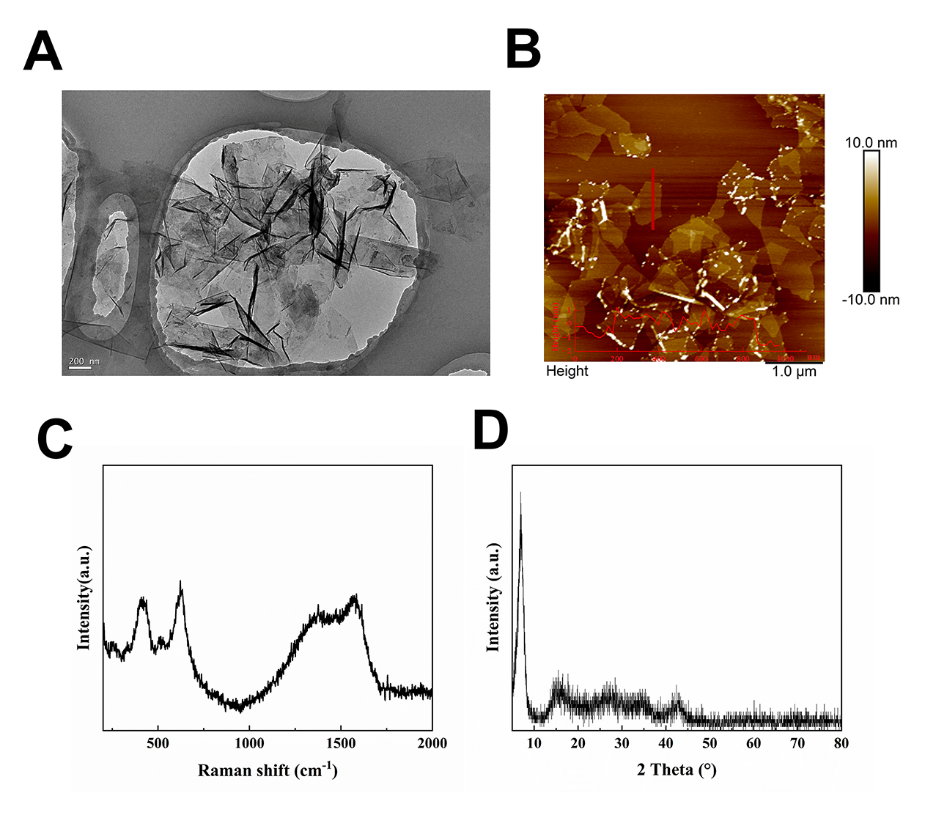
**

**Fig. S1**. Characterization of Ti_3_C_2_ nanosheets. **A** TEM image of single-layer Ti_3_C_2_ nanosheets. **B** AFM image of Ti_3_C_2_ nanosheets. **C** Raman spectrum of Ti_3_C_2_ nanosheets. **D** XRD pattern of Ti_3_C_2_ nanosheets


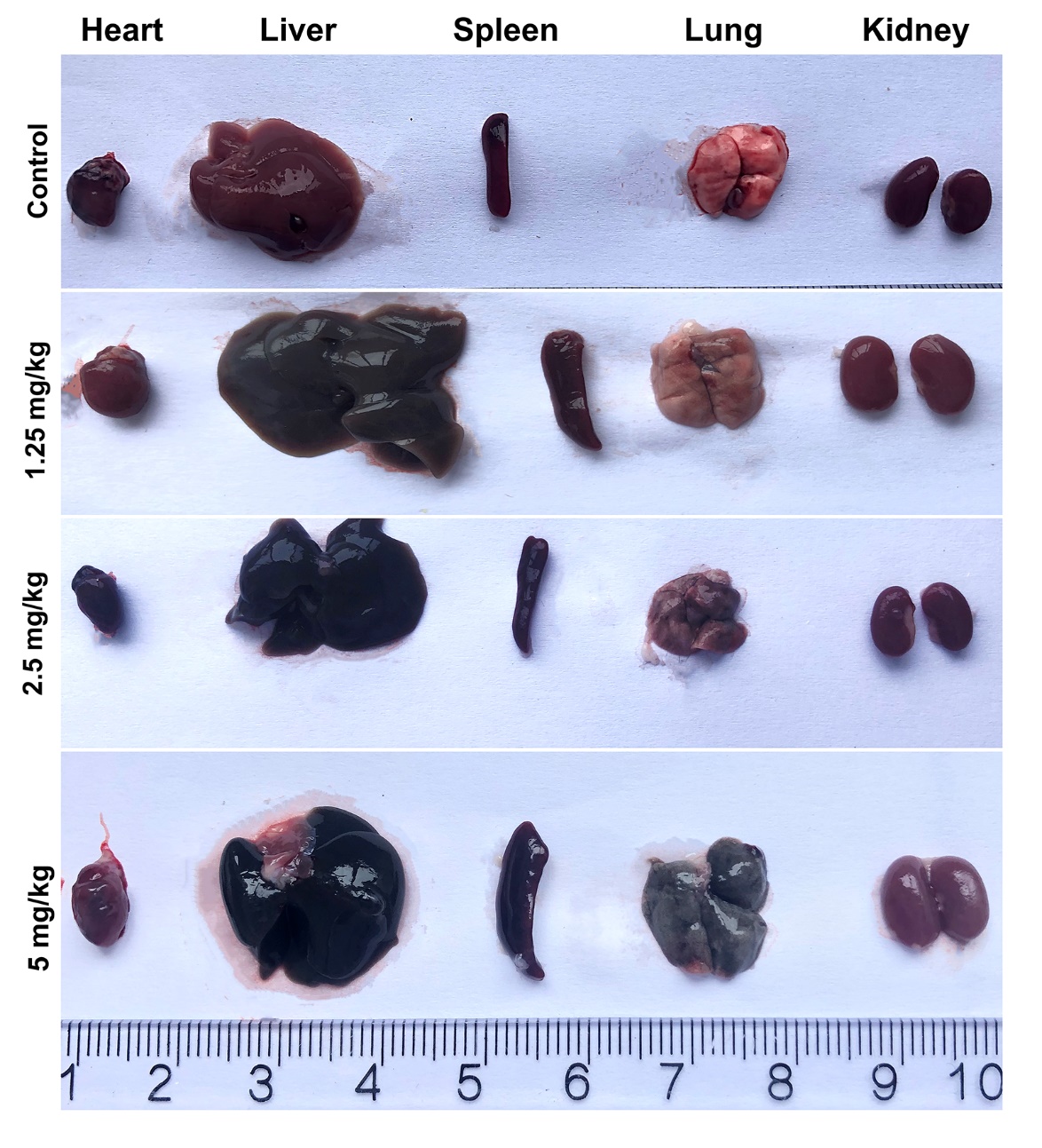


**Fig. S2**. Images of the heart, liver, spleen, lung, and kidney of the mice were collected after intravenous injection of Ti_3_C_2_ nanosheets for three days.

**
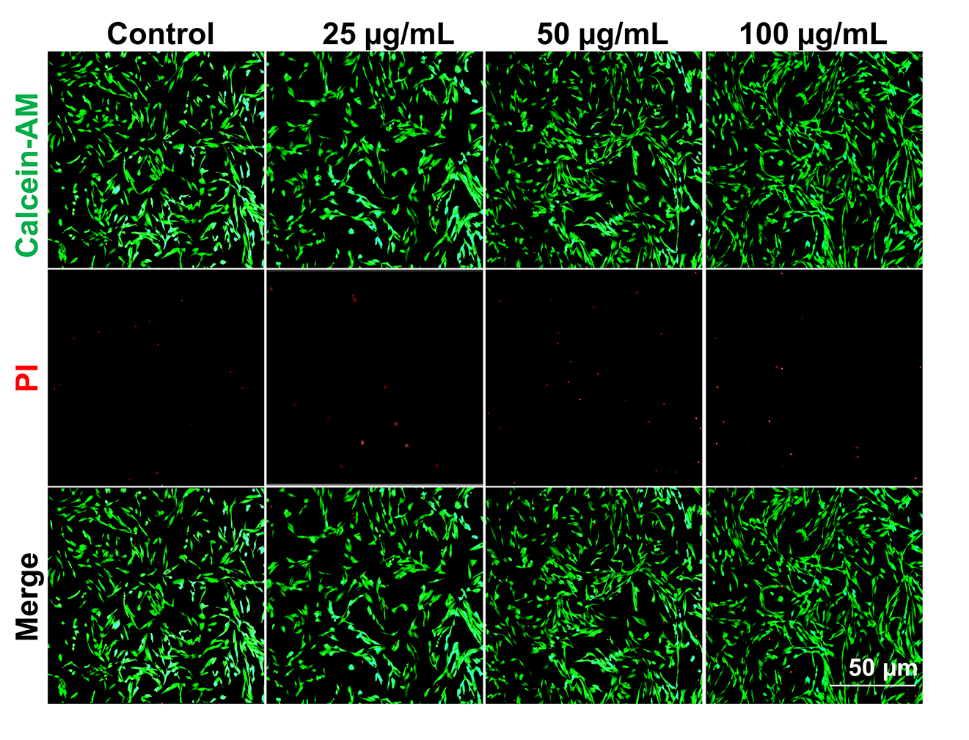
**

**Fig. S3**. Representative images of cells stained with calcein AM/propidium iodide under fluorescence microscope (scale bar = 50 μm).


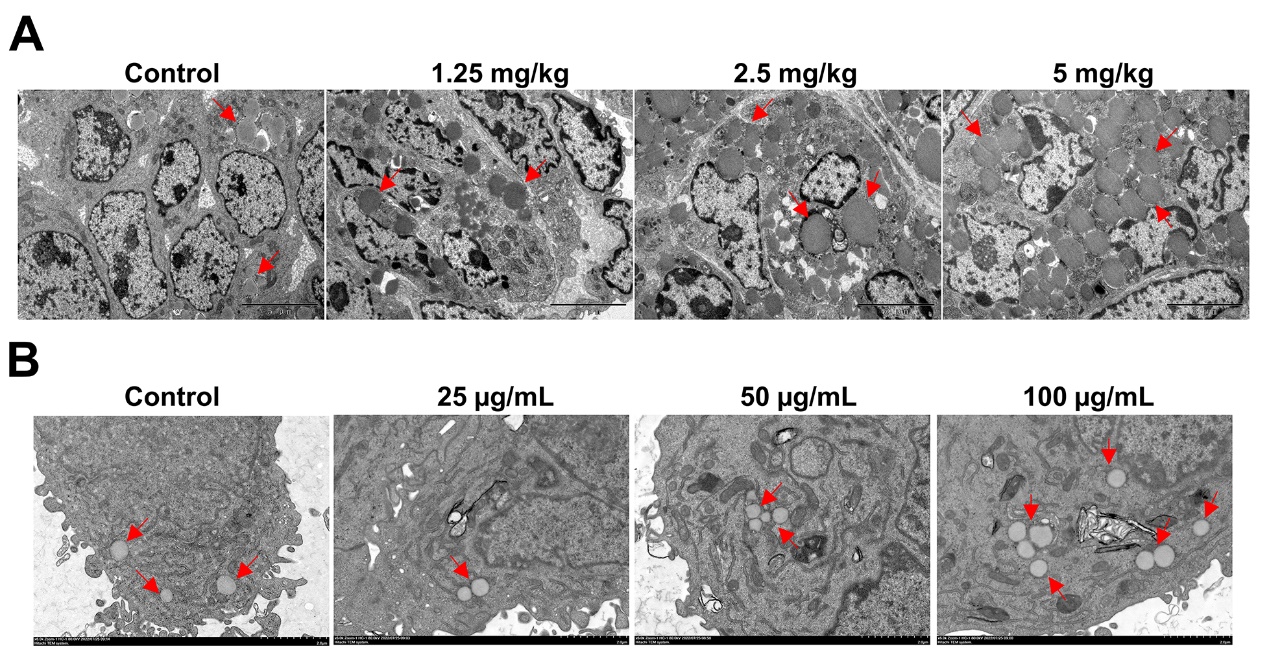


**Fig. S4**. The distribution of lipid droplets in ovarian tissue and KGN cells were observed by TEM (scale bar = 5 μm and 2 μm), The red arrow represents lipid droplets.


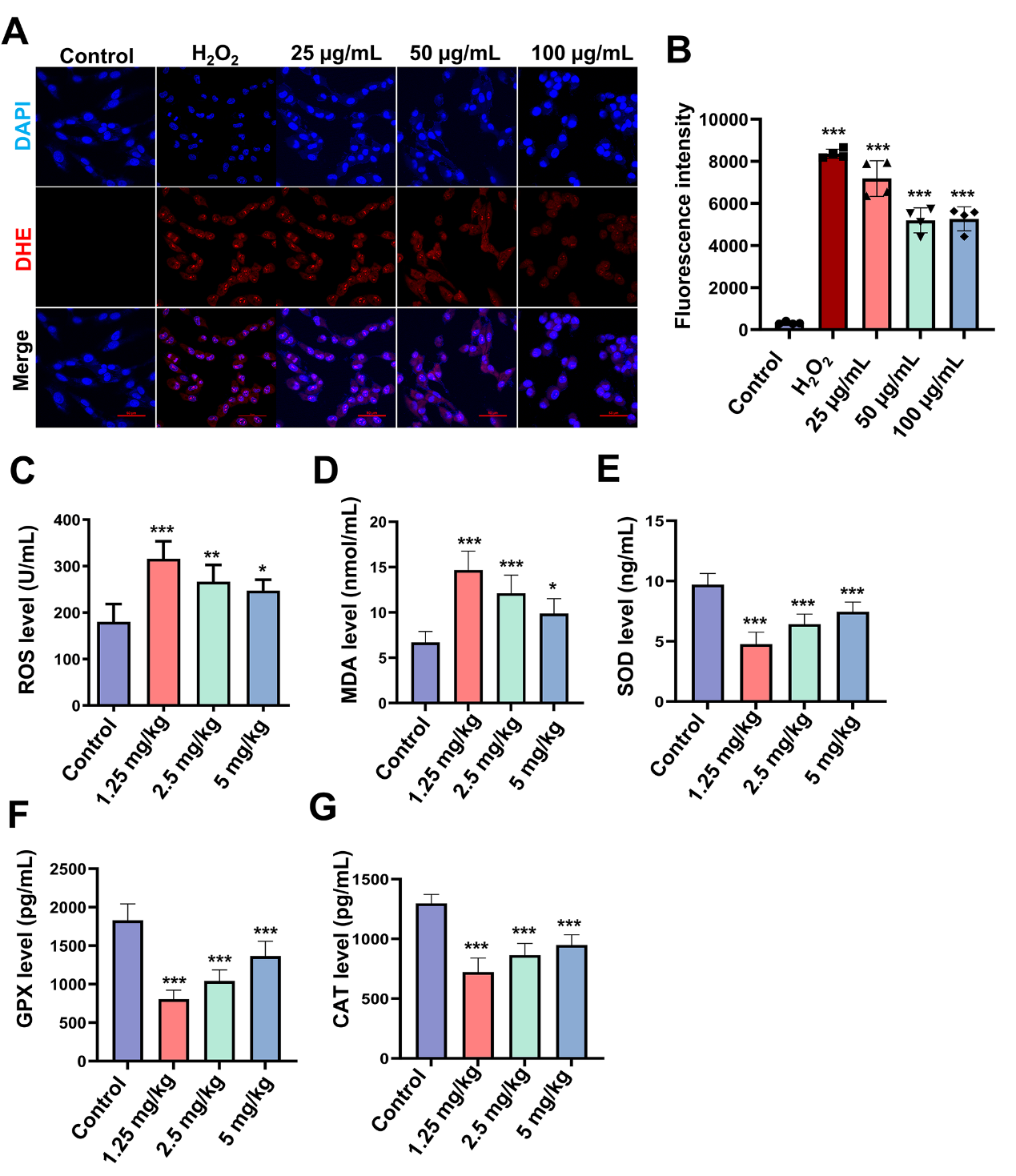


**Fig. S5**. Ti_3_C_2_ nanosheets exposure raised the level of oxidative stress in mouse ovaries and KGN cells. **A, B** Intracellular superoxide anions levels were examined at 24 h after Ti_3_C_2_ nanosheets exposure in KGN cells (scale bars = 50 μm, n = 3 independent repetitions). ROS (**C**), MDA (**D**), SOD (**E**), GPX (**F**) and CAT (**G**) levels were determined from ovaries tissues (n = 3). All data were expressed as means ±standard deviations (**P*<0.05, ***P*<0.01, ****P*<0.001), compared with the control group.
